# Potential of automated image analysis for the measurement of vitiligo lesions

**DOI:** 10.3389/fmed.2025.1623408

**Published:** 2025-08-14

**Authors:** Roberto Mazzetto, Alvise Sernicola, Jacopo Tartaglia, Christian Ciolfi, Mauro Alaibac

**Affiliations:** Dermatology Unit, Department of Medicine (DIMED), University of Padua, Padova, Italy

**Keywords:** vitiligo, dermatology, AI, deep learning, image analysis

## Introduction

Vitiligo is a common, acquired, patchy depigmented skin disease that can be localized or widespread. Its global prevalence ranges from 0.2% to 1.8% (1). Despite its benign nature, the psychosocial burden of vitiligo parallels that of other chronic dermatological diseases such as psoriasis and eczema (2). Lesions, particularly those in visible areas like the face, neck, and hands, contribute to stigma, anxiety, and depression. The chronic nature of vitiligo, combined with the reluctance of many dermatologists to offer active treatment due to pessimism regarding its efficacy, significantly affects patients' quality of life (3). Traditional diagnostic tools such as dermatoscopy, Wood's lamp examination, and skin biopsies continue to play a central role. Dermatoscopy provides detailed insights into pigment changes and structural alterations in vitiligo patches. Wood's lamp is helpful in delineating lesions and assessing disease stability, especially in subjects with light phototypes. Conversely, biopsies are invasive and therefore should be reserved for select cases. Assessing vitiligo disease severity and progression remains challenging due to variability in lesion size, number, distribution, morphology, and pigmentation patterns. In fact, several subjective and semi-objective tools, including the Vitiligo European Task Force assessment (VETFa) and the Vitiligo Area Scoring Index (VASI), have been developed (4, 5), but they are limited by lack of objectivity, consistency, and ease of use in everyday clinical settings (6). Objective methods, such as colorimetry, reflectance confocal microscopy (RCM), and digital image analysis, are promising but often require expensive equipment and specialized expertise (7). Recently, AI and deep learning methods have emerged as powerful tools in medical image analysis, enabling automated pattern recognition, classification, prediction, and decision support. These tools enable objective and reproducible assessment of vitiligo, thereby assisting clinicians in evaluating treatment efficacy and tailoring therapy within a personalized medicine approach.

In this article, we review the principal AI methods currently available to assist clinicians in diagnosing and monitoring vitiligo, evaluating how these tools can integrate into and enhance current clinical practice.

Various machine learning (ML) approaches have been utilized to distinguish between healthy individuals and vitiligo patients and differentiate segmental from non-segmental forms. Recent AI-driven models have enhanced diagnostic capabilities. Hypopigmented dermatoses (HD), a group encompassing vitiligo, pityriasis alba, pityriasis versicolor, and others, pose diagnostic challenges (8). Deep learning has demonstrated higher accuracy rates for vitiligo than for other HD types, attributable to greater sample sizes and better feature learning (9). Studies such as those by Han et al. (10), which achieved 90% accuracy in classifying 12 skin diseases, and Esteva et al. (11), where AI models matched dermatologist-level accuracy in skin cancer detection, highlight their strong diagnostic potential in dermatology, particularly for image-based disease recognition and triage support. Severity evaluation, however, remains crucial for treatment planning. Although VASI is widely used, manual calculation is complicated by irregular lesion shapes. Image analysis software like Photoshop^®^ and AutoCAD^®^ offers improved area measurements but demands significant time and expertise (12). AI models now enable automatic lesion segmentation and colorimetric analysis, streamlining severity assessment (9).

Several deep learning models have been developed for vitiligo analysis with high accuracy and clinical relevance. Convolutional Neural Networks (CNNs) are deep learning architectures designed to automatically learn spatial hierarchies of features from input images. EfficientNet B7-based CNNs have shown strong classification performance, supporting preliminary assessments of vitiligo images (13). Other new CNN-based approaches, such as SE_ResNet-18 and Swin Transformer Large models, have achieved accuracies exceeding 93% (14). Class activation maps generated by these models enhance interpretability, linking model decisions to lesion characteristics and facilitating clinical trust. AU-Net (Attention U-Net), an enhanced AI architecture with integrated attention mechanisms, achieved an estimated accuracy of over 90% in segmenting depigmented areas by selectively focusing on relevant spatial features while reducing background interference (15). EfficientNetV2-L, a CNN optimized for both speed and accuracy, reached 94.5% classification accuracy and demonstrated strong generalizability through cross-validation and external clinical testing (16). AI-based models have also demonstrated high accuracy in vitiligo severity assessment by replicating dermatologist-assigned scores, offering a reliable and objective alternative for clinical evaluation (17). Advanced hybrid AI models using architectures such as YOLO v3 and UNet++ have also achieved reliable morphometric and colorimetric assessments in patients with Fitzpatrick skin types III-IV. Add-on metrics (VAreaA, VAreaR, VColor) correlated well with dermatologist evaluations, making these models suitable for diverse clinical settings (18). In addition to clinical validation, an important consideration is “segmentation,” which refers to the process of partitioning an image into meaningful regions, typically by classifying each pixel, based on visual characteristics such as color, texture, or edges, to isolate and analyze specific structures or objects. Hybrid ViT-CNN leverages the global context modeling of Vision Transformers and the fine-grained spatial details, achieving 96.8% segmentation accuracy (16). Finally, VitiligoNet, a deep CNN framework, offers end-to-end lesion detection, segmentation, and classification, with a reported accuracy of 97.4% that has proven effective across diverse skin tones and clinical scenarios (19). Table 1 compares AI models for vitiligo diagnosis and severity assessment.

**Table 1:** Comparison of AI models for vitiligo diagnosis and severity assessment.

**Model**	**Architecture**	**Accuracy**	**Special features**	**References**
EfficientNet B7	CNN	High	Image classification of vitiligo vs. healthy skin	(13)
SE_ResNet-18	CNN	Lower for non-vitiligo HDs	Lesion area measurement	(9)
Swin transformer large	Transformer-based architecture	93.82%	High interpretability via CAMs	(14)
Double combination (DCNNs + Color Spaces)	ResNet50, VGG16, Xception, Inception v3	87.8%	Combines multiple networks and color spaces	(18)
YOLO v3 + UNet++	Object Detection + Segmentation	92.91%	Simultaneous classification and localization	(18)
Fully convolutional network (FCN) + CNN	CNN modified for segmentation	98.94% (Facial)	Trained on synthetic and internet images	(18)
Modified U-Net + attention (AU-Net)	U-Net with attention mechanism	>90% (estimated)	Accurate segmentation of depigmented areas	(15)
EfficientNetV2-L	CNN	94.5%	Strong performance on cross-validation and external vitiligo datasets	(16)
Hybrid ViT-CNN (ConvNeXt + ViT)	Convolution + transformer hybrid	96.8%	Fuses global ViT features with local CNN features for detailed segmentation	(16)
VitiligoNet	Deep CNN	97.4%	End-to-end lesion detection, segmentation and classification	(19)

Studies differ in dataset size, skin-type mix, lighting, and season; direct comparison is indicative only. CAM, class activation mapping; CNN, convolutional neural network; HD, hypopigmented dermatosis; DCNN, Deep Convolutional Neural Network; YOLO, You Only Look Once (real-time object detection algorithm); UNet++, Nested U-Net architecture for medical image segmentation; FCN, Fully Convolutional Network; ViT, Vision Transformer.

## Discussion

Although dermatology has increasingly embraced AI for various tasks (such as melanoma detection, lesion classification, risk stratification, and dermoscopic image analysis) vitiligo-specific applications are still emerging and progressively expanding (20). Prospective AI developments include training CNN models on images of vitiligo alongside other HDs to improve lesion detection and classification. Such tools could replace Wood's lamp analysis when unavailable. In addition, automated VASI scoring would facilitate remote monitoring, empowering patients to track disease progression and reducing clinical burdens.

Recent research highlights AI's role in predictive analytics. For instance, elevated full blood count parameters (B lymphocyte count and natural killer cell count) could correlate with localized vitiligo, while systemic markers could predict non-segmental disease progression with promising performance (accuracy 73%) (21). This integrative approach is also useful to identify key disease pathways, such as p38 MAPK signaling and oxidative stress modulation, underscoring AI's dual utility in clinical severity scoring and therapeutic target discovery for vitiligo (18, 22). Interpretability remains crucial but visualizing feature maps from neural networks offers insights into internal processing, promoting transparency and fostering clinician confidence. Therefore, AI models could not only provide disease classification labels but also enhance clinical decision-making by offering intuitive, image-based insights that can simplify and complement traditional assessment methods based on clinical variables and scoring systems.

Despite notable progress, most current studies rely on limited datasets and broader validation through multicenter, multiethnic cohorts is needed to overcome this limitation (14). Moreover, models must handle small lesions, subtle repigmentation, and heterogeneous skin tones to ensure robust performance. Ongoing research should focus on larger datasets, multimodal analyses (combining clinical images, laboratory data, and patient-reported outcomes), and real-world validation. Importantly, dermatologists must be actively involved in shaping the ethical and practical frameworks governing AI applications.

Accurate objective measurement is important to classify and standardize the effectiveness of therapeutic agents, especially in the clinical trial setting of new drugs. However, it is known that objective measures provide an incomplete assessment of severity and response to treatment and that subjective patient-reported measures need to be included. For this reason, AI models should not only consider the accuracy of the data collection method but should be trained to integrate the “human” component of daily life and clinical experience which relies on the subjective patient and clinician point of view. This will be particularly useful in the clinical practice to compare the efficacy between treatment options and to tailor the preferred option according to each patient (23).

In fact, even if progress may be slow, disease stabilization and repigmentation are achievable goals, especially when there is a shared understanding between patients and healthcare providers regarding vitiligo and its treatment options (24). The psychological burden associated with the condition is in fact described in various papers that highlight the importance of raising awareness among both physicians and patients about the well-documented relationship between vitiligo and anxiety or other stress-related emotions (25, 26).

The journey toward routine application in everyday clinical settings is restricted by the following issues: regulatory approval under medical device regulations is necessary to allow marketing of image analysis software; compliance to data privacy laws must guarantee informed patient consent and secure use of clinical images that constitute sensitive personal health information; medico-legal concerns related to healthcare provider responsibility, role of clinicians in mitigating the risk of errors and validation in different patient populations still need to be adequately addressed. Finally, practical aspects related to image analysis, such as the scarce or variable contrast that may be associated with low Fitzpatrick phototypes or with seasonal changes in skin color, respectively, may complicate automated interpretation of patient images.

AI is anticipated to be a transformative force in dermatology: from improved diagnostic accuracy to personalized treatment monitoring, AI tools can enhance clinical workflows and patient experiences within a human-centered healthcare model.

With new therapies on the horizon and AI-driven diagnostics becoming a reality, vitiligo is poised to emerge from obscurity into a new era of personalized, technology-enhanced care.
